# Interventions for the Carers of Patients With Eating Disorders

**DOI:** 10.1007/s11920-015-0652-3

**Published:** 2016-01-19

**Authors:** Janet Treasure, Bruno Palazzo Nazar

**Affiliations:** Psychological Medicine, IoPPN, King’s College London (KCL), London, SE5 8AF UK

**Keywords:** Eating disorders, Experienced caregivers helping others (echo), Caregivers, Family intervention, Behaviour change

## Abstract

The aim of this study is to evaluate the recent literature on carers/parenting interventions for people with eating disorders. Interesting and important new findings are highlighted as well as the implications that this may have for treatment. We have reviewed and critically analysed the recent literature. Close others often play an important role in recognising the early signs of eating disorders and accessing and implementing treatment. Their role in helping with recovery is to give support and hold a united front themselves and with the professional team to avoid those common interpersonal reactions that adversely impact on outcome such as accommodating to the illness and reacting with high expressed emotion (overprotection and hostility). Managing this role is difficult, and coping resources are often strained. Carers ask for and are now getting expert training in skills to manage this role. There is an overlap between carer/parenting interventions and family therapies. The interface with close others is critical both for early recognition and access and implementation of treatment. Interventions which equip families and close others with the skills to manage eating disorder behaviours are showing potential at improving outcomes.

## Introduction

An individual with anorexia nervosa (AN) writing about her personal journey said that if she had to describe the illness in one word it would be isolation [1]. The loneliness of an eating disorder [2] can be ameliorated by family members and other carers. However, social problems of the individual her or himself combined with the secondary social problems that arise from abnormal eating behaviours can make support for recovery difficult.

Social factors are both risk and maintaining factors for eating disorders [3]. Individual vulnerabilities in terms of problems in social cognition are found in the acute phase of AN [4] and include deficits in nonverbal emotional expression [5] and sensitivity to threat and to social comparison [6]. In part, these may be a consequence of starvation but a subgroup of patients with AN have social problems that antedate the eating disorder (ED) and remain after recovery [7, 8]. Also, the offspring of people with ED may present anomalies in aspects of social cognition [9]. Thus, problems in social cognition may be an endophenotype that increases the risk of developing an ED.

Patients with bulimia nervosa (BN) experience interpersonal difficulties before the onset of their ED [10] and bulimic behaviours can impair socio-emotional processing [11]. There is limited evidence about social deficits in obesity and binge eating disorder (BED). Individuals with BED experience difficulties of emotional regulation as high as those in AN or BN patients, with high levels of emotional suppression and low levels of emotional reappraisal [12].

The ED itself has a profound interpersonal impact particularly on the family as the age of onset is usually before the individual has left home [13]. Moreover, as the median duration of illness is 6–7 years [14], the passage through developmental milestones is impeded [15]. Many patients remain dependent on their families or the state during their lifetime [16]. Close interpersonal relationships change over the trajectory of the illness and the range of interpersonal skills needed to support recovery vary. The developmental age, work and social adjustment and whether an individual is financially and socially independent have an impact on management. Thus, the impact and role of close others is not simple as it depends on these factors [17].

Caregivers are those who provide care to someone who need supervision or assistance during the course of an illness and hereafter the concept refers to family (parents, spouses) or friends. The reaction of carers to an ED depends on aspects of the illness, the context and the carer themselves and in turn these impact on the course of the illness. In the initial stages, the individual does not recognise that she/he is ill and carers can play an important role by being aware of the early signs and facilitating access to treatment. Expressed emotion is a factor that adversely impacts on the prognosis of many chronic illnesses, and eating disorders are no exception [18]. High expressed emotion is defined as a critical, hostile or overprotective, controlling style of behaviour. Also, family members may collude with ED behaviours, by organising the family around eating disorder rules, ignoring or covering up the negative consequences of the behaviours [19]. These behaviours can cause divisions amongst family members. Some carers shoulder an overly high burden, and others become disempowered from contributing to the management of the illness.

Thus, the social aspect of the illness is important for the wellbeing both of the individual and of the wider network. These two aspects are inter-related; however, interventions often have a separate focus on improving the wellbeing of carers or the patient (as in family-based therapy (FBT)) with the family members providing nutritional support and other aspects of care. The burden on caregivers can be overlooked in the latter role [20].

## The Carer Giving Experience and Carer Coping

Carers express a need for information about the illness [21, 22]. Unfortunately, some literature contains unhelpful, unproved and simplified hypotheses about the families’ role in the illness, which can cause offence and/or guilt [23]. A more helpful aphorism is that ‘Families are the solution and not the problem’.

**Fig. 1:**
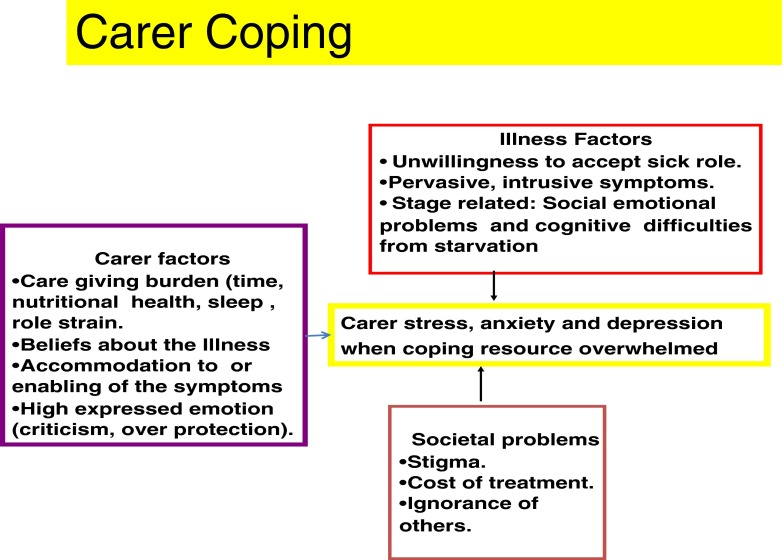
The three main domains that impact on caring

There are many factors that impact on the perceived and actual burden of the caregiving role and how families manage this. The three main domains that impact on caring are the illness itself, the societal reaction to the illness and carer factors, including costs and interactions with services which are illustrated in Fig. 1. Helping the carer to cope with the illness is an essential first step. In the collaborative care model, we use the metaphor of airline safety procedures, in which parents are advised to fix their own oxygen supply first before attending to the child [24].

Aspects of the care giving experience have been summarised in a systematic review [25] which has been recently updated [26]. High perceived burden, and low caregiving efficacy are common and are associated with clinical levels of depression and anxiety [25, 26].

A variety of specific measures have been developed to tap into the relevant problematic areas, and these are shown in Table 1. These include the Eating Disorders Symptom Impact Scale (EDSIS), which measures burden [27]. The Accommodation and Enabling Scale for Eating Disorders (AESED) which measures the tendency of the family to collude with the patient [28, 29]. The Caregiver Skills (CASK) scale measures modifiable aspects of caregiver behaviour that, according to the New Maudsley model, are thought to improve outcomes [30]. The parents versus anorexia nervosa (PvAN) scale measures parental self-efficacy and is based upon the model of FBT [31]. A new measure of carer coping is available to assess coping strategies, called the Family Coping Questionnaire for Eating Disorders (FCQ-ED) [32]. Several of these instruments assess interpersonal means of managing the symptoms, such as avoidance, collusion, confrontation, compassion and connection.

**Table 1:** A summary of the measures that have been used to examine the needs of the carer themselves and the processes used in caregiving

Scale	Domains	Author, year
Carer burden (EDSIS)	• Nutrition (the problem related to low weight and restricted eating) • Guilt (the assumption of responsibility over the illness) • Dysregulated behaviour (e.g. bingeing, alcohol consumption) • Social isolation (for both the family and the individual)	Sepulveda et al. 2008 [23]
Accommodation and Enabling Scale for Eating Disorders (AESED)	• Avoidance and modifying routines • Providing reassurance • Accepting rituals around meals • Turning a blind eye to unwanted behaviours and allowing family functioning to be controlled	Sepulveda et al. 2009 [24]
Caregiver skills (CASK)	• Bigger picture (the ability to take the long view and not get caught up in the details of the illness) • Self-care (strategies to improve carers own mood and resilience) • Biting-your-tongue (not getting caught up in nagging and bickering about the illness) • Insight and acceptance (the ability to recognise symptoms as part of the illness and to not personalise the behaviours) • Emotional intelligence (the ability to regulate emotional reactions despite being provoked and to have empathy for the other) • Frustration tolerance (to be able to withhold getting drawn into conflict about aspects of the illness)	Hibbs et al. 2015 [25, 26]
Parents versus anorexia nervosa (PvAN)	• Perceptions of the relative influence of parents compared with the anorexia over the child • Acknowledgement of the possession of knowledge and strategies for bringing about recovery • Parental ability to privilege their own expertise and instincts above those of professionals • Parental view that the task of recovery is theirs rather than that of their child • Parental ability to stand up to anorexia despite distress caused for their child • Parental ability to act now in standing up to anorexia rather than become entangled in searching for how they might have caused it	Rhodes et al. 2005 [27]
Carer coping	Five subscales regarding coping mechanisms: • Avoidance • Coercion • Collusion • Information • Positive communication with the patient	Fiorillo et al. 2014 [28]

Legend: this table illustrates the domains of caregiving that have been examined in caring for people with eating disorders

Eating disorder symptoms are pervasive and intrusive into the family life, and interpersonal relationships become entangled with the disorder in a complex manner. The patients’ primary and secondary difficulties in social cognition can make these relationships even more difficult. For example, the reduced level of facial expressivity in ED patients [5, 33] makes it hard for others to appreciate their level of terror. ED patients can be impervious to the impact on close others [34] with the overt life-threatening nature of the symptoms contrasting with the individuals own unwillingness to accept the sick role.

Carers themselves have their own practical and emotional reaction to the illness, which for the most part is helpful but can impede recovery if there is division within the family, collusion with some aspects of the illness and high expressed emotion. Siblings can develop their own problems or leave home prematurely as their needs may be neglected [35]. Carers’ distress and means of coping with the caring role may add to the distress of the individual with an ED, and a vicious circle develops [36]. Poor coping lead to distress, and this, in turn, increases the tendency to either overprotect the individual and accommodate to the illness or enter into unproductive fights. Many of the elements of carer stress and coping are universal, and various models have been described [37]. Models of stress have been described for caregivers of other illnesses such as Alzheimer’s disease where carer coping style is determined by environmental stress and resources available [38].

Most research has focused on the caregiving for AN, there is little evidence about BN families and hardly any about BED. Caregivers of BN and BED report high levels of depression, anxiety and stress [39].

### Change Processes and Techniques

This involves sharing information with carers about how to (1) increase carer coping, (2) effective support and change skills and (3) reduce unhelpful interpersonal behaviours.

1. Models of carer coping.

Social support increases carer coping [40–42], but high levels of contact time reduces resilience [17, 40]. Thus, mothers and partners often have higher levels of burden and distress [41]. Coping may be more difficult if carers have their own eating disorder [43]. Maladaptive coping, expressed emotion and carer needs predicted later carer burden [44].

No matter what the stage of illness, carers ask for information to help with their caregiving role [21, 22, 45]. It is developmentally appropriate for carers of young people to provide meal support whereas this may be less appropriate in the case of adults with a severe enduring illness. Nonetheless, carers are drawn in to provide emotional and financial support in the context of the many problems that can arise with this chronic illness. Moreover, carers play an important role in bridging the isolation and can be actively involved in treatments to ameliorate the secondary consequences [46].

A systematic review of interventions primarily targeting the needs of caregivers and increasing their coping abilities found benefits in terms of reduced burden and distress [47]. However, the quality of many of the studies was limited; the descriptions of the intervention were poorly detailed, and the change processes involved were unclear. Most of the studies focused on carer outcomes only.

2. Skills to manage the illness.

A key part of FBT is empowering the parents to provide nutritional support, and the success of this aspect of therapy is marked by an improvement in weight in the early phase [48, 49]. This can be difficult particularly if the illness has been present for some time when families may resort to critical and controlling strategies [50]. A new adaptation to family-based treatment has a focus on increasing carer skills [51•] to deal with behaviours that have become more entrenched and appears to have benefits.

The New Maudsley collaborative care approach was developed particularly to help families with prolonged illness [24]. This model teaches carers skills such as positive communication using motivational interviewing (MI), meal support and the management of other difficult behaviours. Lay and professional carers can be adequately trained to deliver these MI interventions [52]. This approach has been given in a group format [53] and also in a scalable form with a carer self-management format. DVDs (http://www.succeedfoundation.org) and books for carers describe various behaviour change strategies and how social support can be used to enhance refeeding and other eating disorder symptoms [54, 55]. Moreover, there is evidence that adding an intervention for carers can improve patient outcome, reduce service use and carer burden [56•]. Both carers and patients comment on the positive changes such as increased understanding and more communion following this form of intervention [57–59].

Interventions that target other socio-emotional aspects of eating disorders [60, 61] and cognitive rigidity have also been developed [62]. Some of those suggest adaptations of novel psychological techniques with standard family interventions for ED but these warrant further investigation. An adaptation of acceptance and commitment therapy [60] integrating FBT skills (re-nourishment exposure to feared foods and situations facilitated by parents) for families of adolescent AN patients has found changes in psychological acceptance of the disorder for all family members and a half of participants attaining full remission. An integration of skills from dialectic behavioural therapy (daily review of symptom change; analysis of behaviour chains; development of crisis plans; emotional regulation skills for the whole family) with FBT (improvement of communications during mealtimes) [63] for families of adolescent bulimia nervosa has also been tried [64]. Other interventions such as cognitive remediation therapy (CRT) focus on AN thinking styles which might impair adherence and outcome of psychological therapies. A CRT self-help model delivered in collaboration of carers showed good acceptability and is also an intervention in which carers role might be important [62].

3. Reducing unhelpful behaviours—interpersonal maintaining patterns.

The cognitive interpersonal model of Schmidt and Treasure (2006) describes how interpersonal processes are impacted by the visibility of AN [65], which can elicit overprotection or conflict [66•]. Carer accommodating behaviour is higher in the early phase of illness and is associated with both patient and carer distress (Rhind, submitted). Carers’ distress and carers’ expressed emotion (overprotection, criticism and hostility) are similar at all stages of illness [67]. The New Maudsley model of collaborative care reduces both accommodation and expressed emotion [67] and improves patient outcomes [56•].

## Conclusion

Interventions for caregivers need to take into account the stage of illness and whether interpersonal maintaining behaviours such as accommodation, expressed emotion or family division are present. A variety of psychoeducational interventions can improve carer coping. FBT successfully teaches skills to manage behaviours present in the early phase of the illness. The New Maudsley approach addresses some of the maintaining interpersonal behaviours. New interventions specifically targeting partners [68] have been produced, and a consideration of the needs of siblings may also be of value. Most of this work has focused on anorexia nervosa, and more work is needed to understand the needs of close others in relationship to bulimia nervosa and binge eating disorder. This remains a work-in-progress, but the interim conclusions are that the psychosocial and interpersonal aspects of eating disorders play an important role in their management.
